# Supplementary material for: Site-specific analysis of the SARS-CoV-2 glycan shield
Source: bioRxiv. 2020 Mar 28:2020.03.26.010322. Preprint. [Version 1] doi: 10.1101/2020.03.26.010322 (PMC7239077; doi:10.1101/2020.03.26.010322)
Supplement: Supplement 1 [file media-1.docx]

Supplementary Information for:

**Site-specific analysis of the SARS-CoV-2 glycan shield**

Yasunori Watanabe^1,2,3#^, Joel D. Allen^1#^, Daniel Wrapp^4^, Jason S. McLellan^4^, Max Crispin^1*^

^1^ School of Biological Sciences, University of Southampton, Southampton, SO17 1BJ, UK

^2^ Oxford Glycobiology Institute, Department of Biochemistry, University of Oxford, South Parks Road, Oxford OX1 3QU, UK

^3^ Division of Structural Biology, University of Oxford, Wellcome Centre for Human Genetics, Oxford, OX3 7BN, UK

^4^ Department of Molecular Biosciences, The University of Texas at Austin, Austin, TX 78712, USA

^#^ These authors contributed to this work equally.

^*^To whom correspondence may be addressed. Email: max.crispin@soton.ac.uk

This document includes supplementary table 1 and supplementary table 1 legend

**Supplementary Table 1. Glycoform abundances observed across SARS CoV-2 S protein.** The upper table shows the categorized glycan compositions at each N-linked glycan site, with the global averages shown in the right-hand table. The lower table further categorizes the glycan compositions into oligomannose-, hybrid-, and complex-type
